# Genetic analysis of the *Arabidopsis* protein kinases* MAP3Kε1 *and *MAP3Kε2* indicates roles in cell expansion and embryo development

**DOI:** 10.3389/fpls.2012.00228

**Published:** 2012-10-10

**Authors:** Suraphon Chaiwongsar, Allison K. Strohm, Shih-Heng Su, Patrick J. Krysan

**Affiliations:** ^1^Faculty of Science and Agricultural Technology, Rajamangala University of Technology LannaChiang Mai, Thailand; ^2^Department of Genetics, University of Wisconsin at MadisonMadison, WI, USA; ^3^Department of Horticulture and Genome Center of Wisconsin, University of Wisconsin at MadisonMadison, WI, USA

**Keywords:** MAP kinase, MAP3Kε1, MAP3Kε2, cell expansion, embryo development, *Arabidopsis*

## Abstract

*MAP3Kε1* and *MAP3Kε2 *are a pair of *Arabidopsis thaliana* genes that encode protein kinases related to cdc7p from *Saccharomyces cerevisiae*. We have previously shown that the *map3kε1;map3kε2* double-mutant combination causes pollen lethality. In this study, we have used an ethanol-inducible promoter construct to rescue this lethal phenotype and create *map3kε1*^-/-^;*map3kε2*^-/-^ double-mutant plants in order to examine the function of these genes in the sporophyte. These rescued double-mutant plants carry a yellow fluorescent protein (*YFP*)*-MAP3Kε1* transgene under the control of the alcohol-inducible *AlcA* promoter from *Aspergillus nidulans*. The double-mutant plants were significantly smaller and had shorter roots than wild-type when grown in the absence of ethanol treatment. Microscopic analysis indicated that cell elongation was reduced in the roots of the double-mutant plants and cell expansion was reduced in rosette leaves. Treatment with ethanol to induce expression of *YFP-MAP3Kε1* largely rescued the leaf phenotypes. The double-mutant combination also caused embryos to arrest in the early stages of development. Through the use of YFP reporter constructs we determined that *MAP3Kε1* and *MAP3Kε2* are expressed during embryo development, and also in root tissue. Our results indicate that *MAP3Kε1* and *MAP3Kε2 *have roles outside of pollen development and that these genes affect several aspects of sporophyte development.

## INTRODUCTION

The *Arabidopsis *genes *MAP3Kε1* and *MAP3Kε2* encode protein kinases that were originally named as members of the MAP kinase kinase kinase (MAP3K) gene family (MAPK Group, 2002). More recent analysis has indicated that MAP3Kε1 and MAP3Kε2 are more closely related to cdc7p from *Schizosaccharomyces pombe* and Cdc15p from *Saccharomyces cerevisiae*, which are not MAP3Ks (Jouannic et al., 2001; Champion et al., 2004a,b). Cdc7p is one of the components of the septation initiation network (SIN). The SIN has been shown to regulate the formation of the septum in fission yeast after chromosome segregation has been completed (Simanis, 2003). The functionally equivalent pathway in the budding yeast *S. cerevisiae* is called the mitotic exit network (MEN), and it regulates cytokinesis and mitotic exit (Simanis, 2003). SIN-like elements such as sid1p-, cdc16-, and mob1p-related proteins have been identified in both the *Arabidopsis *and**rice (*Oryza sativa*) genomes (Bedhomme et al., 2008), leading to the speculation that a SIN-like pathway may exist in plants.

The *Arabidopsis* genome encodes the following signaling proteins closely related to the core elements of the SIN pathway: *AtMAP3Kε1, AtMAP3Kε2, AtSGP1, AtSGP2, AtMAP4Kα1, *and *AtMAP4Kα2* (Champion et al., 2004b; Bedhomme et al., 2008). These *Arabidopsis* genes partially rescue homologous fission yeast mutants and encode proteins that interact with their fission yeast counterparts. There is currently, however, no direct evidence in support of the idea that plants use these SIN-related components to regulate cytokinesis (Champion et al., 2004b; Bedhomme et al., 2008).

We have previously reported that single-mutant plants carrying T-DNA null alleles for either *map3kε1* or *map3kε2* displayed no obvious abnormal phenotypes, while the double-mutant combination caused pollen lethality (Chaiwongsar et al., 2006). These results suggested that functional compensation could be occurring between *MAP3Kε1* and *MAP3Kε2* such that the loss of one gene is compensated for by the second gene (Liljegren et al., 2000). This appears to be the case for pollen development, but remained an untested hypothesis for the sporophyte since the pollen-lethality of the double-mutant combination prevents the formation of a homozygous double-mutant sporophyte. It has been reported that *MAP3Kε1* is expressed in all organs of the plant, and that *MAP3Kε1* and *MAP3Kε2* are expressed strongly in regions of the sporophyte that contain actively dividing cells (Jouannic et al., 2001; Chaiwongsar et al., 2006; Bedhomme et al., 2009). These reports indicate that *MAP3Kε1* and *MAP3Kε2* are likely to have functions outside of pollen development.

In order to generate *map3kε1*^-/-^;*map3kε2*^-/-^ double-mutant plants we made use of an alcohol-inducible transcription system to rescue the pollen lethal phenotype of the *map3kε1;map3kε2* double-mutant combination. This system is derived from the filamentous fungus *Aspergillus nidulans* and has been successfully adopted for use in plants (Caddick et al., 1998; Roslan et al., 2001; Laufs et al., 2003). It is composed of the *alcR*-encoded transcriptional activator (AlcR) and a promoter derived from the *alcA* promoter (Kulmburg et al., 1992). In *A. nidulans*, these elements allow for the controlled activation of a series of structural genes important for alcohol catabolism (Pickett et al., 1987). In our study we used the *alcR/alcA* system to express**yellow fluorescent protein (*YFP*)*-MAP3Kε1* in *map3kε1;map3kε2* double-mutant pollen, thereby allowing for the generation of *map3kε1*^-/-^;*map3kε2*^-/-^ plants. We report here on our phenotypic analysis of these rescued *map3kε1*^-/-^;*map3kε2*^-/-^ double-mutant plants. Taken together our genetic analyses indicated that *MAP3Kε1* and *MAP3Kε2* are involved in multiple aspects of plant development, including root cell elongation, rosette leaf expansion, and embryo deve- lopment.

## RESULTS

### USING AN ALCOHOL-INDUCIBLE PROMOTER CONSTRUCT TO PRODUCE RESCUED *map*3kε1^-/-^;*map*3kε2^-/-^ DOUBLE-MUTANT PLANTS

We have previously described the T-DNA null alleles *map3kε1 *and *map3kε2* (Chaiwongsar et al., 2006). In that previous work we observed that homozygous single-mutant lines for either *map3kε1 *or *map3kε2* did not display any obvious abnormal phenotypes when grown under standard laboratory conditions. We also found that the *map3kε1;map3kε2 *double-mutant combination caused pollen lethality (Chaiwongsar et al., 2006). These results suggested that there is functional redundancy between *MAP3Kε1* and *MAP3Kε2* during pollen development. Because *map3kε1;map3kε2 *pollen is non-viable, however, it is not straightforward to generate a plant that is homozygous double-mutant for these T-DNA null alleles. For this reason one cannot directly explore via genetic analysis the function of this pair of potentially redundant genes in the sporophyte.

In order to overcome this challenge we needed an experimental strategy that would allow *map3kε1;map3kε2 *double-mutant pollen to be viable, which would in turn allow a homozygous double-mutant sporophyte to be formed. The strategy that we chose was to place the wild-type *MAP3Kε1* coding region under the transcriptional control of an alcohol-inducible promoter system and induce expression of *MAP3Kε1* during pollen and embryo development so that these processes could proceed normally. After seed had been produced by these plants, the progeny could be grown under conditions that do not induce expression of the *MAP3Kε1* transgenic construct, thereby allowing us to observe any phenotypic consequences of the reduced levels of *MAP3Kε1* and *MAP3Kε2*.

**FIGURE 1:**
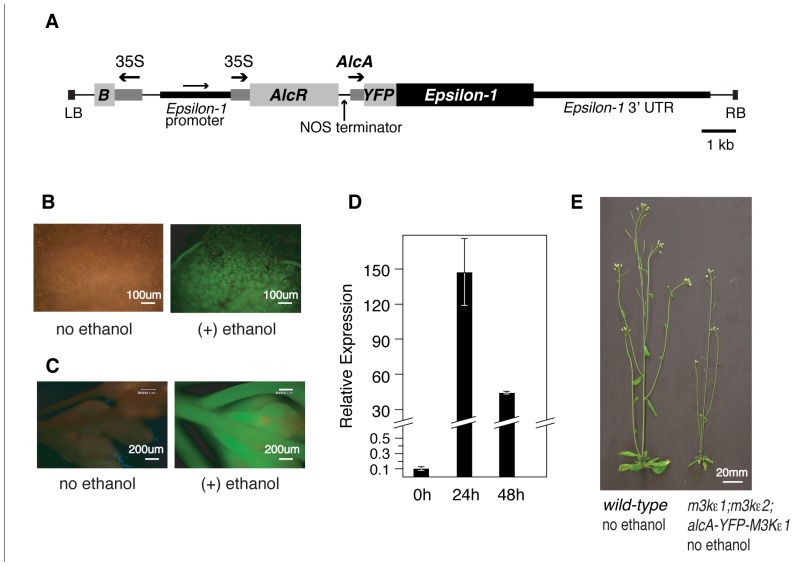
** Using an alcohol-inducible promoter construct to generate *map3kε1*^-/-^*;map3kε2*^-/-^ double-mutant plants**. **(A)** Plasmid map of the T-DNA portion of the alcohol-inducible *YFP-MAP3Kε1* expression construct. Arrows indicate the direction of transcription for each of the promoters. The *MAP3Kε1* native promoter is present on the construct as an artifact of the cloning procedures used to assemble this plasmid. “B” indicates the coding region for the *Bar* gene that serves as a plant-selectable marker by encoding resistance to the herbicide Basta. “LB” and “RB” indicate the T-DNA left border and right border sequences. “35S” indicates the cauliflower mosaic virus 35S promoter. “AlcA” indicates the alcohol-inducible promoter element. Scale bar indicates 1 kb. **(B,C)** Epifluorescence images of a rosette leaf (**B) **or an inflorescence (**C)** of an *map3kε1*^-/-^*;map3kε2*^-/-^;*alc*-*YFP-MAP3Kε1* plant before and after exposure to exogenous ethanol. Images were collected 24 h after ethanol treatment. Green color indicates YFP fluorescence. Red color is due to autofluorescence of the plant tissue. **(D)** Two-week-old *map3kε1*^-/-^*;*map3kε2^-/-^;*alc*-*YFP-MAP3Kε1 *seedlings carrying the *alcA:YFP-MAP3Kε1* construct were sprayed with ethanol to induce *YFP-MAP3Kε1* expression. RNA was isolated at the indicated time points. “Relative expression” indicates the RNA level of *YFP-MAP3Kε1* transcripts relative to *MAP3Kε1* in wild-type control seedlings not carrying the *alcA:YFP-MAP3Kε1* construct. Standard deviation is based on three independent replicates of real-time quantitative RT-PCR. Expression of *YFP-MAP3Kε1* the zero-hour time point prior to ethanol treatment was at a level of 0.1 compared to wild-type *MAP3Kε1* in control seedlings. **(E)** Representative 3-week-old wild-type Columbia and *map3kε1*^-/-^*;map3kε2*^-/-^;*alc*-*YFP-MAP3Kε1* plants grown in soil without exposure to exogenous ethanol.

We began by constructing a plasmid in which a YFP tag is translationally fused to the wild-type MAP3Kε1 coding sequence (CDS) under the transcriptional control of the ethanol-inducible *alcA* promoter (**Figure 1A**; Caddick et al., 1998; Roslan et al., 2001; Deveaux et al., 2003). We included the YFP tag on this construct so that we could monitor YFP-MAP3Kε1 expression at the protein level via fluorescence microscopy. This construct was then introduced via *Agrobacterium*-mediated transformation into plants that were homozygous for *map3kε2* and heterozygous for *map3kε1*. PCR-based genotyping was used to identify *map3kε1*^-^^/^^+^;*map3kε2*^-/-^ primary transformants that were also carrying the *alcA:YFP-MAP3Kε1* construct. In order to determine if these plants carried a functional copy of the *alcA:YFP-MAP3Kε1* construct, we monitored YFP-MAP3Kε1 protein expression using fluorescence microscopy. Twenty-four hours after treatment with a 1% ethanol spray, strong induction of YFP fluorescence was observed throughout the aerial tissue of the plants, including leaves and flowers (**Figures 1B,C**), indicating that the *alcA:YFP-MAP3Kε1* construct present in these lines was performing as expected.

In order to rescue the lethal phenotype displayed by *map3kε1;map3kε2* double-mutant pollen, developing flowers of *map3kε1*^-^^/^^+^;*map3kε2*^-/-^;alcA:YFP-MAP3Kε1 plants were sprayed with 1% ethanol on a daily basis throughout their life-cycle in order to activate expression of *alcA:YFP-MAP3Kε1* and allow double-mutant pollen grains to survive. Progeny from these ethanol-treated plants were genotyped and several *map3kε1*^-/-^;*map3kε2*^-/-^;alcA:YFP-MAP3Kε1 individuals were identified. These results indicated that the *alcA:YFP-MAP3Kε1* construct was able to rescue the *map3kε1;map3kε2* pollen lethal phenotype, and that the YFP tag did not interfere with MAP3Kε1 function during pollen development.

Progeny derived from eight independently transformed *map3kε1*^-/-^;*map3kε2*^-/-^;*alc*-*YFP-MAP3Kε1* lines were screened using quantitative reverse-transcriptase PCR to find the line with the lowest background level of *YFP-MAP3Kε1* expression. In the absence of ethanol treatment, the double-mutant line chosen for phenotypic analysis in this study expressed *YFP-MAP3Kε1* at a level that was 10-fold lower than that of native *MAP3Kε1 *expressed in wild-type plants (**Figure 1D**). When treated with exogenous ethanol, seedlings carrying this construct displayed strong induction of the *YFP-MAP3Kε1* gene up to a level ca. 150 times higher than that of native *MAP3Kε1* expressed in wild-type plants(**Figure 1D**). When grown in soil in the absence of exogenous ethanol treatment, progeny of this *map3kε1*^-/-^;*map3kε2*^-/-^;*alc*-*YFP-MAP3Kε1* line had an overall reduction in the size of their rosette leaves and bolts (**Figure 1E**).

### THE REDUCED LEAF SIZE OF *map*3kε1^-/-^;*map3kε2*^-/-^;*alc*-*YFP-MAP3K*ε1 PLANTS IS PARTIALLY RESCUED BY ETHANOL TREATMENT

**FIGURE 2:**
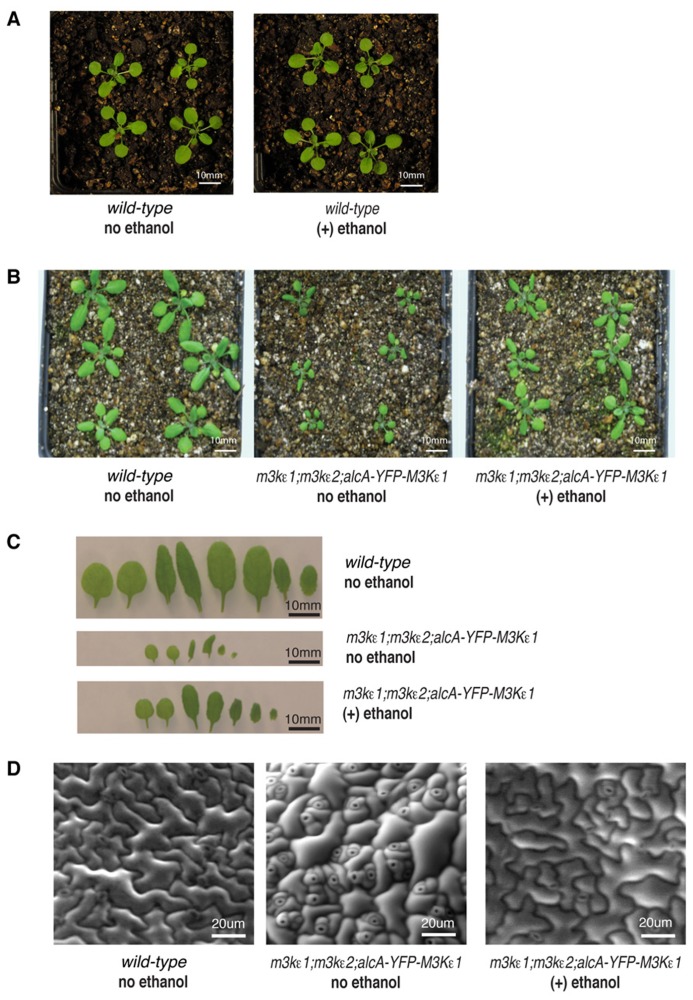
**Reduced cell expansion in rosette leaves of *map3kε1*^-/-^;*map3kε2^-/-^;alc-YFP-MAP3Kε1* plants**. **(A,B)** Fourteen-day-old soil-grown plants. **(C)** Detached rosette leaves from 14-day-old soil grown plants arranged in developmental order from left to right. **(D)** Environmental scanning electron microscope images of leaf epidermal cells from 14-day-old soil grown plants. For ethanol treatment, plants were sprayed with 1% ethanol (v/v) every 2 days throughout development. “no ethanol” samples were sprayed on the same time schedule with distilled water.

In order to determine if the reduced size of rosette leaves seen in *map3kε1*^-/-^;*map3kε2*^-/-^;*alc*-*YFP-MAP3Kε1* plants grown in the absence of ethanol was due to the low level of *MAP3Kε1* expression in these plants relative to wild-type, we performed the following experiment. Two pots of *map3kε1*^-/-^;*map3kε2*^-/-^;*alc*-*YFP-MAP3Kε1* plants were grown side-by-side, along with wild-type controls. One pot of *map3kε1*^-/-^;*map3kε2*^-/-^;*alc*-*YFP-MAP3Kε1* plants was sprayed with 1% ethanol every 2 days throughout development, while the second was sprayed with water only. We also subjected wild-type Columbia plants to the same regimen of water and ethanol treatments to determine if ethanol treatment had any effect on the growth of wild-type plants. As seen in **Figure 2A**, treatment with ethanol did not affect the growth of wild-type plants. Spraying *map3kε1*^-/-^;*map3kε2*^-/-^;*alc*-*YFP-MAP3Kε1* plants with ethanol to induce *YFP-MAP3Kε1* expression, however, resulted the partial rescue of the reduced-leaf-size phenotype (**Figures 2B,C**).

We also performed scanning electron microscopy of the leaf epidermis of *map3kε1*^-/-^;*map3kε2*^-/-^;*alc*-*YFP-MAP3Kε1* plants to observe the size and shape of the epidermal cells. This analysis revealed that the epidermal cells of untreated *map3kε1*^-/-^;*map3kε2*^-/-^;*alc*-*YFP-MAP3Kε1* plants were heterogeneous in size, with a majority of the cells being much smaller than those of wild-type (**Figure 2D**). Some relatively large cells were also observed in the untreated mutant lines, but these cells did not have the typical jig-saw shape of wild-type epidermal cells. These results indicate that *MAP3Kε1/2* may be important for the normal expansion of leaf epidermal cells. The cellular phenotypes displayed by the untreated *map3kε1*^-/-^;*map3kε2*^-/-^;*alc*-*YFP-MAP3Kε1* plants were partially rescued when the plants were sprayed with ethanol every 2 days throughout development to induce expression of YFP-MAP3Kε1 (**Figure 2C**).

### ROOT LENGTH IS REDUCED IN *map*3kε1^-/-^;*map3kε2*^-/-^;*alc*-*YFP-MAP3Kε1* PLANTS

**FIGURE 3:**
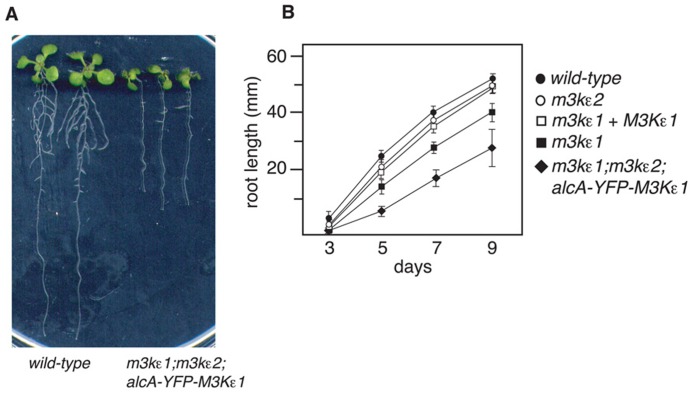
**Reduced root length of *map3kε1*^-/-^;*map3kε2*^-/-^; alc-YFP-MAP3Kε1 plants** **(A)** Nine-day-old plants grown on vertically oriented agar plates. **(B)** Primary root length of seedlings grown on agar plates. “days” indicates age of seedling. *map3kε1*+*MAP3Kε1* indicates a line that is homozygous for the *map3kε1* mutation and also carries an ectopic copy of the wild-type *MAP3Kε1* locus. Mean root length is reported. *n* ≥ 30 for each genotype. Error bars indicate standard error.

To investigate the role of *MAP3Kε1/2 *in root growth, seeds of the *map3kε1*^-/-^;*map3kε2*^-/-^;*alc*-*YFP-MAP3Kε1* mutant line as well as a wild-type control were sown on agar plates containing growth media. Root length was then measured after 5 days of growth on vertically oriented plates. We observed that the primary roots of the *map3kε1*^-/-^;*map3kε2*^-/-^;*alc*-*YFP-MAP3Kε1* plants were ca. 40% shorter than wild-type (**Figure 3A**). In order to further investigate the contribution of *map3kε1* and *map3kε2* to this phenotype, we performed a time-course experiment to measure root length over the course of 9 days of growth on vertical agar plates. As additional controls, this experiment included *map3kε1*and *map3kε2* homozygous single-mutant lines as well as the *map3kε1*^-/-^;*map3kε2*^-/-^;*alc*-*YFP- MAP3Kε1* line.

As shown in **Figure 3B**, the primary roots of *map3kε1* homozygous single-mutant lines were significantly shorter than those of wild-type. This phenotype is fully rescued by the presence of an ectopic copy of the wild-type *MAP3Kε1* genomic locus (**Figure 3B**). By comparison, the roots of *map3kε1*^-/-^;*map3kε2*^-/-^;*alc*-*YFP-MAP3Kε1* plants were found to be even shorter than those of the *map3kε1* single-mutant.

We next attempted to use ethanol-induction to rescue the short-root phenotype of *map3kε1*^-/-^;*map3kε2*^-/-^;*alc*-*YFP-MAP3Kε1* plants. We tested a variety of ethanol-treatment procedures but were not able to generate substantial *YFP-MAP3Kε1 *expression in root tissue as determined by fluorescent microscopy, and no phenotypic rescue was observed.

### REDUCED CELL EXPANSION IN *map*3kε1^-/-^;*map3kε2*^-/-^;*alc*-*YFP-MAP3Kε1* ROOTS

**FIGURE 4:**
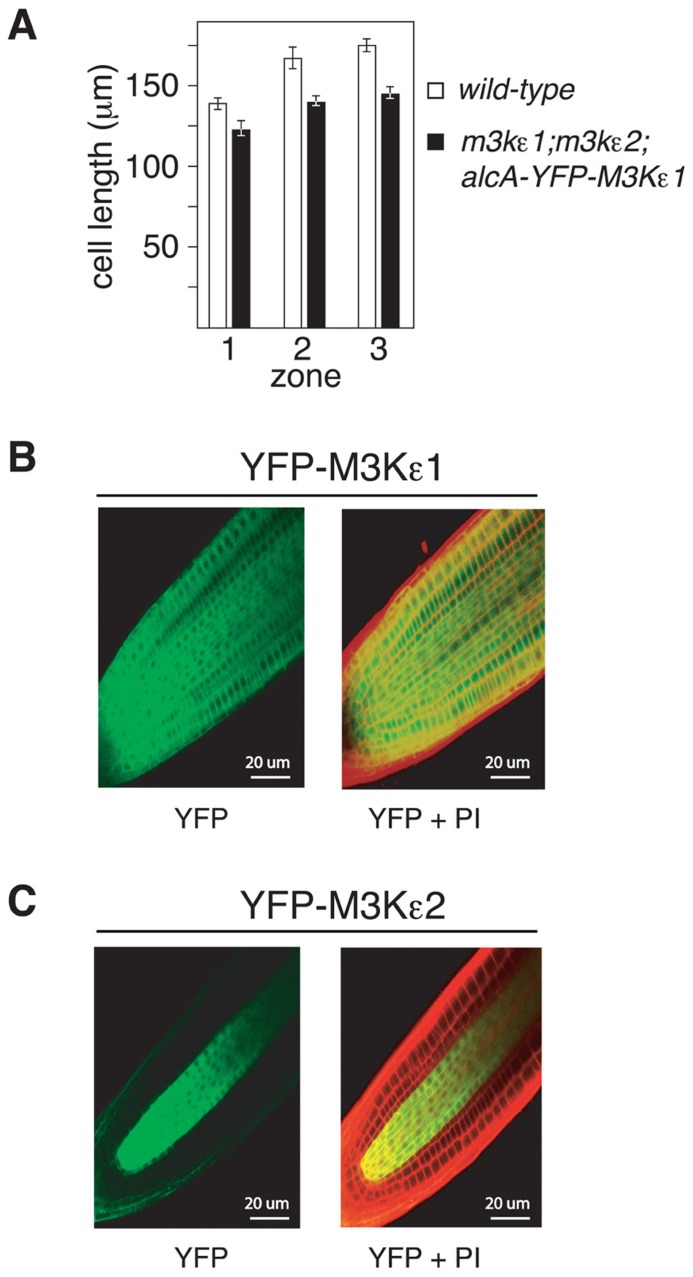
**Reduced cell elongation in the roots of *map3kε1*^-/-^; map3kε2^-/-^;alc-YFP-MAP3Kε1 plants**. **(A)** Mean length of root epidermal cells from three previously defined zones of root growth (Cruz-Ramirez et al., 2004). Zone 1 is the root meristem, zone 2 is the cell elongation zone, and zone 3 is the mature zone where cell elongation has ceased. *n* ≥ 15. Error bars represent the standard error. **(B,C)** Confocal microscopy images of primary root tips of transgenic lines expressing YFP-MAP3Kε1 or YFP-MAP3Kε2 from their respective native promoters. Roots were stained with propidium iodide (PI) to highlight cell walls (indicated by red color in the images). Left panels display YFP fluorescence in green. Right panels are merged images of the YFP and PI fluorescent signals. Emission was detected at 590–640 nm for PI and 500–550 nm for YFP.

To further explore the cause of the short-root phenotype displayed by *map3kε1*^-/-^;*map3kε2*^-/-^;*alc*-*YFP-MAP3Kε1* plants, epidermal cell length was measured in three different zones of 7-day-old roots. These zones correspond to the root meristem, the elonga- tion zone, and the mature zone where cell elongation has ceased (Cruz-Ramirez et al., 2004). Root epidermal cells of *map3kε1*^-/-^; map3kε2^-/-^;*alc*-*YFP-MAP3Kε1* seedlings were shorter than wild-type in all of these zones. On average the root epidermal cells were ca. 20% shorter in the *map3kε1*^-/-^;*map3kε2*^-/-^;*alc*-*YFP-MAP3Kε1* plants when compared to wild-type, indicating that cell elongation is reduced (**Figure 4A**). Because the overall length of the primary root in *map3kε1*^-/-^;*map3kε2*^-/-^;*alc*-*YFP-MAP3Kε1* plants is ca. 40% less than wild-type (**Figure 3**), reduced cell elongation alone cannot fully account for the total reduction in root length. The mutant lines must also have fewer total cells in the primary root, suggesting a reduction in the rate of new cell production in the roots of *map3kε1*^-/-^;*map3kε2*^-/-^;*alc*-*YFP-MAP3Kε1* plants.

We also documented the expression pattern of MAP3Kε1 and MAP3Kε2 in *Arabidopsis* primary roots using YFP fusion constructs. *YFP-MAP3Kε1 *and *YFP-MAP3Kε2* translational fusions under the transcriptional control of their respective native promoters were introduced into wild-type *Arabidopsis* plants via *Agrobacterium*-mediated transformation. Transgenic lines were generated and fluorescence microscopy was used to detect YFP-MAP3Kε1 or YFP-MAP3Kε2 expression. After seed germination, YFP-MAP3Kε1 was strongly expressed throughout the root apical meristem (**Figure 4B**). By contrast, YFP-MAP3Kε2 expression in the root apical meristem was largely restricted to vascular tissues (**Figure 4C**). These results indicate that MAP3Kε1 and MAP3Kε2 are both expressed in the root apical meristem region with overlapping, but distinct expression patterns.

### THE *map3kε1*^-/-^;*map3kε2*^-/-^ DOUBLE-MUTANT COMBINATION CAUSES EMBRYO LETHALITY

**FIGURE 5:**
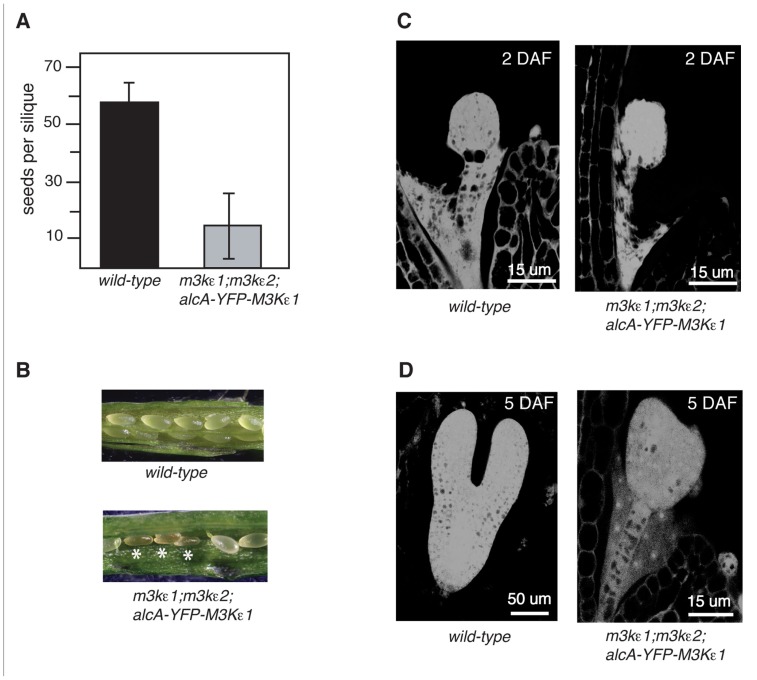
** Seed production and embryo development of *map3kε1*^-/-^;*map3kε2*^-/-^;*alc*-*YFP-MAP3Kε1* plants**. **(A)** Mean number of seeds produced per silique for plants grown in the absence of exogenous ethanol treatment. Error bars indicate standard error. *n* ≥150 siliques for each genotype. **(B)** Siliques of 3-week-old plants grown in the absence of exogenous ethanol treatment. Shriveled seeds are indicated by white asterisks. **(C,D)** Microscopic analysis of embryo development. **(C)** Images at 2 days after flowering (DAF). **(D)** Images at 5 DAF. Images were obtained by capturing the autofluorescence of fixed embryos using a confocal microscope.

*map3kε1*^-/-^;*map3kε2*^-/-^;*alc*-*YFP-MAP3Kε1* plants produce fewer seeds than wild-type when grown in the absence of exogenous ethanol (**Figure 5B**). The mutant plants produce an average of 15 seeds per silique, compared to 55 seeds per silique for wild-type (**Figure 5B**). Although *map3kε1;map3kε2* pollen is not viable (Chaiwongsar et al., 2006), Alexander’s staining showed that pollen from *map3kε1*^-/-^;*map3kε2*^-/-^;*alc*-*YFP-MAP3Kε1* plants was viable in the absence of exogenous ethanol treatment, suggesting that the *alcA:YFP-MAP3Kε1* construct is expressed in the absence of ethanol treatment in developing pollen (data not shown). This phenomenon might be due to the fact that the ethanolic fermentation pathway is normally activated during pollen development (Mellema et al., 2002), resulting in the production of acetaldehyde and ethanol, which can both act as inducers of the *alcA* promoter (Tadege and Kuhlemeier, 1997).

We have previously shown that* map3kε1;map3kε2* double-mutant female gametophytes develop normally (Chaiwongsar et al., 2006). Therefore, the decrease in seed production of the *map3kε1*^-/-^;*map3kε2*^-/-^;*alc*-*YFP-MAP3Kε1* plants may be due to a disruption of seed development, rather than reduced pollen fitness. To test this possibility, *map3kε1*^-/-^;*map3kε2*^-/-^;*alc*-*YFP-MAP3Kε1* plants were grown and allowed to self-pollinate in the absence of exogenous ethanol treatment. Developing seeds from the *map3kε1*^-/-^;*map3kε2*^-/-^;*alc*-*YFP-MAP3Kε1* plants were then analyzed microscopically. Siliques from wild-type and *map3kε1*^-/-^;*map3kε2*^-/-^;*alc*-*YFP-MAP3Kε1* plants were removed from the main shoot and compared. During normal *Arabidopsis* seed development, the external appearance of immature seeds changes from white to green, and then to brown at ca. 15–17 days after flowering (DAF). At 7 DAF, we observed that a portion of the seeds from the *map3kε1*^-/-^;*map3kε2*^-/-^;*alc*-*YFP-MAP3Kε1* plants were smaller than wild-type and brown (**Figure 5A**). By 16 DAF, a majority of the seeds from *map3kε1*^-/-^;*map3kε2*^-/-^;*alc*-*YFP-MAP3Kε1* siliques appeared collapsed and shriveled (data not shown).

Immature seeds within a single wild-type silique normally develop at approximately the same rate (Sparkes et al., 2003). In wild-type plants grown in our laboratory conditions, the embryos reached the globular stage by 2 DAF and the torpedo stage by 5–6 DAF (**Figures 5C,D**). However, many of the embryos from *map3kε1*^-/-^;*map3kε2*^-/-^;*alc*-*YFP-MAP3Kε1* plants grown under the same conditions displayed delayed development. All of the *map3kε1*^-/-^;*map3kε2*^-/-^;*alc*-*YFP-MAP3Kε1* embryos reached the globular stage at the normal time of 2 DAF, but at 6 DAF development was stalled at the globular or transition stage in many of the embryos (**Figure 5D**). At 7 DAF, most of the *map3kε1*^-/-^;*map3kε2*^-/-^;*alc*-*YFP-MAP3Kε1 *seeds had collapsed and the embryos were arrested at either the globular or heart stage (data not shown).

### *MAP3K*ε1 AND *MAP3K*ε2 ARE EXPRESSED DURING EMBRYOGENESIS

**FIGURE 6:**
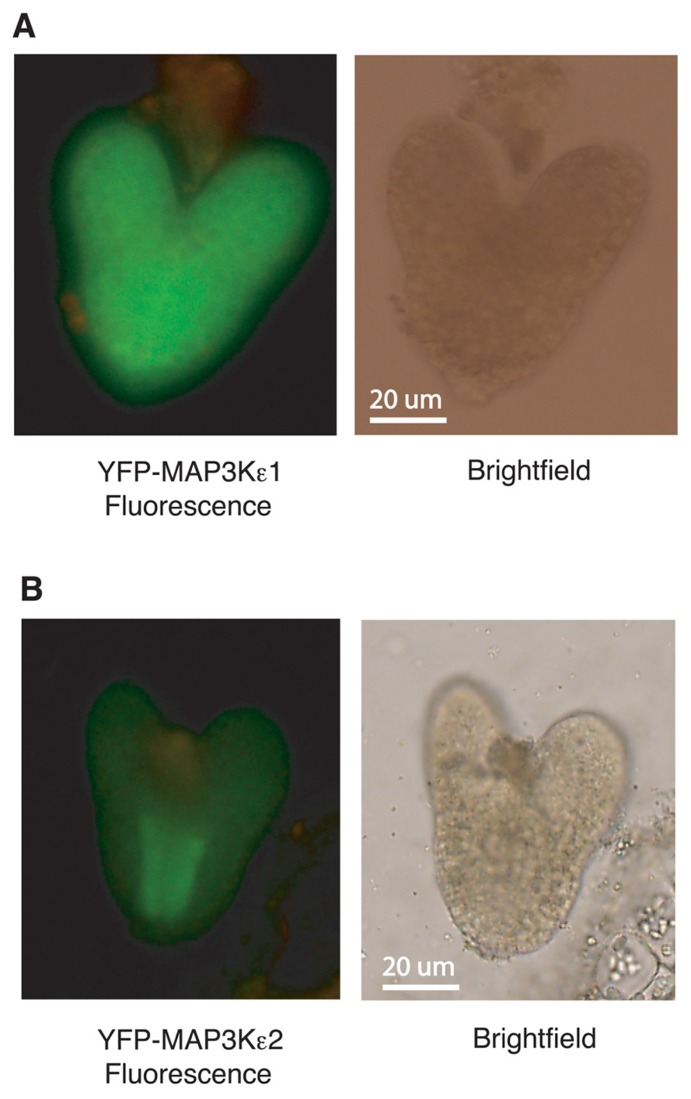
***MAP3Kε1* and *MAP3Kε2* are expressed during embryo development.** **(A,B)** Embryos from wild-type plants carrying a *YFP-*MAP3Kε1 construct or a *YFP-MAP3Kε2* construct under the transcriptional control of their respective native promoters were removed from seeds and observed using epifluorescence microscopy to detect YFP-MAP3Kε1 and YFP-MAP3Kε2 protein expression. Green color represents YFP-MAP3Kε1 or YFP-MAP3Kε2 expression. Brightfield and fluorescence images of each embryo are shown.

The arrest of embryo development observed with *map3kε1*^-/-^;*map3kε2*^-/-^;*alc*-*YFP-MAP3Kε1* plants suggested that *MAP3Kε1*, *MAP3Kε2 *play an important role in normal embryo development. If these proteins are directly involved in embryo development, then one would expect the proteins to be expressed in developing embryos. To directly explore this question we used the *YFP-MAP3Kε1 *and *YFP-MAP3Kε2*, translational fusion constructs described above to determine if these proteins are expressed in developing embryos. These constructs express the fusion proteins using the gene’s respective native promoters. YFP fluorescence was detected via fluorescence microscopy in the developing embryos of plants expressing YFP-MAP3Kε1 and YFP-MAP3Kε2 (**Figure 6**). YFP-MAP3Kε1 expression was observed throughout all the tissues of the embryo. By contrast, the expression pattern of YFP-MAP3Kε2 was noticeably concentrated in the developing vascular cells (**Figure 6**).

## DISCUSSION

We have previously shown that the* map3k*ε*1*^-/-^;map3kε*2*^-/-^ double-mutant combination causes pollen lethality (Chaiwongsar et al., 2006). In order to study the function of MAP3Kε1/2 in the sporophyte, we used an ethanol-inducible**system to provide conditional expression of *MAP3Kε1* during pollen development, thereby rescuing the *map3kε1;map3kε2* pollen lethality and allowing us to generate *map3k*ε*1*^-/-^;map3kε*2*^-/-^ double-mutant plants. Using this system, YFP-MAP3Kε1 expression could be strongly induced by spraying plants with a solution of 1% ethanol. It should be noted that the basal level of expression from the *alc*-*YFP-MAP3Kε1* construct in the absence of exogenous ethanol treatment was not zero. The *map3kε1*^-/-^;*map3kε2*^-/-^;*alc*-*YFP-MAP3Kε1* line that we chose for detailed study expressed *YFP-MAP3Kε1* at a level that was 10% that of the wild-type *MAP3Kε1* gene in wild-type plants. The presence of detectable transcription from the *alcA* promoter in transgenic plants in the absence of exogenous inducer is commonly observed and is possibly caused by the production of an endogenous inducer in tissue where the oxygen level is low (Roberts et al., 2005).

The residual expression of *YFP-MAP3Kε1* by the *alc* promoter that we observed in our experiments highlights some of the advantages and disadvantages of using the *alc* promoter to rescue pollen lethal mutations. Residual expression can be seen as a disadvantage of this system because it prevents one from obtaining a true null allele for the gene of interest, so one cannot observe the effect of the complete loss of a protein on the growth and development of the plant. Residual expression could also, however, be seen as an advantage of the system. In cases where the complete absence of a gene product causes embryo lethality, residual expression from the *alc* promoter may be able to provide enough gene product to allow the embryo to develop to maturity so that the effect of the partial loss of the gene product of interest can be evaluated in developing seedlings and plants. In the case of our study, the residual expression of *MAP3Kε1* driven by the *alc *promoter in the absence of ethanol was sufficient to allow seedling germination and plant growth, but cell expansion in roots and leaves was compromised. The leaky expression of the *alc* promoter therefore allows one to produce what is effectively a “weak” allele of a locus for which a null allele would otherwise be lethal. If a true null allele is desired, then it may be possible to use other inducible expression systems such as the dexamethasone-inducible system, the beta-estradiol-inducible system, or a heat shock-inducible promoter in order to obtain a lower background level of gene expression (Borghi, 2010). Alternatively, a pollen-specific promoter (Twell et al., 1990) could be used to rescue the pollen-lethal mutation during pollen development, thus allowing for the formation of a homozygous-mutant embryo. If the basal expression level of the pollen-specific promoter in other stages of plant development was sufficiently low, then one would be able to observe the effect of loss of that gene product during embryo development and beyond.

*map3kε1*^-/-^;*map3kε2*^-/-^;*alc*-*YFP-MAP3Kε1* plants were able to germinate and grow in the absence of ethanol treatment. We observed that these *map3kε1*^-/-^;*map3kε2*^-/-^;*alc*-*YFP-MAP3Kε1* plants had reduced primary root cell elongation and reduced epidermal leaf cell expansion. This reduced cell expansion may explain the overall small size of the mutant plants. The induction of YFP-MAP3Kε1 expression in these plants via exogenous ethanol treatment could partially rescue the leaf phenotypes, indicating that the *map3kε1* mutation in the *map3kε2 *background is responsible for these phenotypes.

The short-root phenotype of *map3kε1*^-/-^;*map3kε2*^-/-^;*alc*-*YFP-MAP3Kε1* plants could not be rescued by exogenous ethanol treatment. Despite the application of various concentrations of exogenous ethanol and various methods of ethanol application, including direct application or exposure of plants to ethanol vapor, we could not detect substantial expression of YFP-MAP3Kε1 in root tissue, even though YFP-MAP3Kε1 expression was strongly induced in the leaves of these same plants (data not shown). These results suggest that the ethanol-inducible construct used in these experiments may not be suitable for providing inducible gene expression in root tissue.

We have previously shown that MAP3Kε1 is localized to the plasma membrane (Chaiwongsar et al., 2006), suggesting that MAP3Kε1 may be involved in the process of cell expansion by performing an as of yet unknown function at the plasma membrane. This view is supported by the abnormality of the plasma membrane in *map3k*ε*1;map3k*ε*2* double-mutant pollen (Chaiwongsar et al., 2006). In addition, the thicker intine layer observed with *map3kε1;map3kε2*double-mutant pollen suggests that MAP3Kε1 may also affect cell wall synthesis. Future studies will be needed to pinpoint the specific aspect of cell expansion that is influenced by MAP3Kε1 and MAP3Kε2.

Using fluorescence microscopy and YFP-tagged fusion proteins, we observed that MAP3Kε1 and MAP3Kε2 were expressed in the embryos. A majority of the embryos that develop on *map3kε1*^-/-^;*map3kε2*^-/-^;*alc*-*YFP-MAP3Kε1* plants arrest their development at the globular or heart stages. Because *map3kε1*^-/-^;*map3kε2*^-/-^;*alc*-*YFP-MAP3Kε1 *plants have a low basal level of transcription from the *alcA:YFP-MAP3Kε1* construct, it seems likely that viable seeds are able to form on *map3kε1*^-/-^;*map3kε2*^-/-^;*alc*-*YFP-MAP3Kε1 *plants as a result of slightly higher *alc*-*YFP-MAP3Kε1* activity in a given developing embryo. We frequently observed that the first siliques that develop on *map3kε1*^-/-^;*map3kε2*^-/-^;*alc*-*YFP-MAP3Kε1* plants yield more viable seeds than later siliques, but it is not clear what the mechanism responsible for this differential seed yield is.

In roots the expression of *MAP3Kε2* seems to be focused in the area of vascular tissue, while *MAP3Kε1* is more uniformly distributed across all tissue types. In addition, we observed that *map3kε1*^-/-^ single-mutant plants have shorter roots than wild-type, whereas *map3kε2*^-/-^ single-mutants are indistinguishable from wild-type. These results suggest that *MAP3Kε1 *can compensate for the absence of *MAP3Kε2*, while the converse is not true. The embryo-lethality observed in *map3kε1*^-/-^;*map3kε2*^-/-^;*alc*-*YFP-MAP3Kε1 *plants indicates that absence of both gene products is lethal, suggesting substantial functional redundancy within this pair of genes. One interesting question raised by these results is how the expression of *MAP3Kε2* in the vascular tissue of roots is able to compensate for the absence of *MAP3Kε1* in the non-vascular tissues of *map3kε1*^-/-^ single-mutant plants. It is possible that a low level of *MAP3Kε2 *expression occurs outside of vascular tissue that is below the level of detection afforded by the visualization of YFP fluorescence. Alternatively, *MAP3Kε2* expression could be induced in non-vascular tissues in the *map3kε1*^-/-^ mutant background. Further study will be required to determine the extent to which *MAP3Kε1* and *MAP3Kε2* have unique versus redundant roles in plant growth and development.

In this study we have demonstrated how an ethanol-inducible promoter system can be used to rescue a pollen-lethal mutation, thereby allowing one to study mutations in the sporophyte that would otherwise be lethal. Our initial analysis of the resulting *map3kε1*^-/-^;*map3kε2*^-/-^ double-mutant plants indicated a role for these kinases in the processes of cell elongation and embryo development. Further studies will be needed to pinpoint the specific pathways in which MAP3Kε1 and MAP3Kε2 function. The work reported here provides a starting point for more detailed functional analysis of this pair of genes and also provides an example of the utility of the alcohol-inducible system for studying pollen-lethal mutations.

## MATERIALS AND METHODS

### PLANT GROWTH CONDITIONS AND MUTANT ALLELES

*Arabidopsis thaliana* (Columbia ecotype) were grown under continuous light at 22–25°C in soil. To activate expression of the alcohol-inducible *alcA*-*YFP-MAP3Kε1* construct in plants growing in soil, the plants were sprayed with 1% (v/v) ethanol every 2 days throughout development.

### PLASMID CONSTRUCTS

In order to create the *YFP-MAP3Kε1* fusion construct, a wild-type *MAP3Kε1* genomic clone previously described (Chaiwongsar et al., 2006) was modified using site-directed mutagenesis to add recognition sites for the restriction enzymes *Avr*II and *Age*I immediately after the start codon. The YFP CDS was then PCR amplified from a plasmid vector using PCR primers that added an *Nhe*I site to the 5′. end of the CDS and an *Age*I site to the 3′. end. This PCR-amplified fragment containing the *YFP* CDS was then ligated to the modified *MAP3Kε1* clone using sticky ends generated by *Avr*II, *Age*I, and *Nhe*I cleavage. *Nhe*I sticky ends are identical to those produced by *Avr*II. The resulting construct contains the *YFP* CDS fused in frame to the 5′. end of the *MAP3Kε1* coding region and retains the *MAP3Kε1* native promoter. The same strategy was used to construct the *YFP-MAP3Kε2* fusion construct. The resulting plasmids were introduced into plants using *Agrobacterium*-mediated transformation (Clough and Bent, 1998).

In order to construct the ethanol-inducible version of the *YFP*-*MAP3Kε1 *fusion protein, recognition sites for the restriction enzyme *Rsr*II were introduced immediately downstream of the ATG start codon of the *YFP*-*MAP3Kε1* coding region described above. A 3.7 kb fragment from the plasmid pBinSRNACATN (Caddick et al., 1998) was then PCR amplified using primers that added *Rsr*II sites to the PCR product and cloned into the *Rsr*II sites in *YFP*-*MAP3Kε1*. This 3.7 kb fragment contained an *AlcR* expression cassette as well as the *AlcA* promoter. The resulting plasmid contains the *YFP*-*MAP3Kε1* gene under the transcriptional control of the *AlcA* promoter (**Figure 1A**). This vector was introduced into *Arabidopsis* plants via *Agrobacterium*-mediated transformation (Clough and Bent, 1998).

### AGAR PLATE SEEDLING GROWTH ASSAYS

Seeds were surface sterilized with 95% (v/v) ethanol for 5 min, air dried, and sown on 0.5× Murashige and Skoog basal salt mixture media (pH 5.8; Sigma) with 0.7% agar (w/v). After sowing, seeds were allowed to imbibe in the dark at 4°C for 2 days and were then grown on vertically oriented plates under constant light at 22–24°C. To measure the root lengths, images were captured using a flat-bed scanner and analyzed with Adobe Illustrator software.

### MICROSCOPY

Confocal microscopy was used to collect images of root epidermal cells to measure cell length. For these experiments, cell walls were stained using propidium iodide (PI) and optical sections were obtained using a Zeiss LSM 510 Meta Confocal microscope (Zeiss, Thornwood, NY, USA). For PI staining, fresh plant tissue was transferred to a solution of 10 µg/mL of PI (Sigma) for 15–30 min. Imaging was performed using a 568-nm excitation line and an emission window of 585–610-nm. Epifluorescence images were captured digitally using an Olympus DP70 camera (Center Valley, PA, USA) attached to an Olympus BX60 epifluorescence microscope (Center Valley, PA, USA).

To detect YFP fluorescence in embryos, embryos were removed from the seed coats at various stages and viewed with an epifluorescence microscope. To study embryo development, embryos were fixed with 4% glutaraldehyde (in 12.5 mM cacodylate, pH 6.9) overnight. After fixation, the embryos were dehydrated through a 20, 40, 60, 80, and 100% ethanol series for 20 min per step. After dehydration, embryos were cleared in 1:1 (v/v) benzyl benzoate:benzyl alcohol for minimum of 2 h. Embryos were then mounted with glycerol and observed with a Zeiss LSM 510 Meta Confocal microscope with a 488-nm argon laser and an LP539 filter to detect autofluorescence.

### QUANTITATIVE RT-PCR

In order to measure the expression level of *YFP-MAP3Kε1 *expression in the *map3kε1*^-/-^;*map3kε2*^-/-^;*alc*-*YFP-MAP3Kε1* lines, total RNA was isolated from rosette leaf tissue using a Qiagen RNeasy Mini Kit (Qiagen, Valencia, CA, USA). First-strand cDNA was prepared using 100 µg of total RNA with the Super Script First-Strand synthesis system (Invitrogen, Carlsbad, CA, USA). The resulting first-strand cDNA was diluted 1:100 with water and used as a template for real-time, quantitative RT-PCR amplification using an iCYCLER PCR system (BioRad, Hercules, CA, USA). SYBR Green (Molecular Probes, Eugene, OR, USA) was used to detect RT-PCR product accumulation. Primers for the actin gene *ACT2* were used as a control (Genbank Accession: U41998). To detect *MAP3Kε1* expression, the predicted cDNA sequence of *MAP3Kε1* was used to design the following pair of PCR primers: ε1-RT-A1, 5′.-AAAAACATTGTGAAGTATCTTGGGTCGTC-3′.; ε1-RT-A2, 5′.-GCTTCTTTACGAATTTCGCGAGAACGATC-3′. (Chaiwongsar et al., 2006).

## Conflict of Interest Statement

The authors declare that the research was conducted in the absence of any commercial or financial relationships that could be construed as a potential conflict of interest.

## References

[B1] Bedhomme M. Jouannic S. Champion A. Simanis V. Henry Y. (2008). Plants, MEN and SIN. *Plant Physiol. Biochem.* 46 1–10 18053736 10.1016/j.plaphy.2007.10.010

[B2] Bedhomme M. Mathieu C. Pulido A. Henry Y. Bergounioux C. (2009). *Arabidopsis* monomeric G proteins, markers of early and late events in cell differentiation. *Int. J. Dev. Biol.* 53 177–185 19123141 10.1387/ijdb.072488mb

[B3] Borghi L. (2010). Inducible gene expression systems for plants. *Methods Mol. Biol.* 655 65–75.. 20734254 10.1007/978-1-60761-765-5_5

[B4] Caddick M. X. Greenland A. J. Jepson I. Krause K. P. Qu N. Riddell K. V. (1998). An ethanol inducible gene switch for plants used to manipulate carbon metabolism. *Nat. Biotechnol.* 16 177–180 9487526 10.1038/nbt0298-177

[B5] Chaiwongsar S. Otegui M. S. Jester P. J. Monson S. S. Krysan P. J. (2006). The protein kinase genes MAP3Kepsilon1 and MAP3Kepsilon2 are required for pollen viability in *Arabidopsis thaliana*. *Plant J.* 48 193–205 16965555 10.1111/j.1365-313X.2006.02863.x

[B6] Champion A. Picaud A. Henry Y. (2004a). Reassessing the MAP3K and MAP4K relationships. *Trends Plant Sci.* 9 123–129 15003235 10.1016/j.tplants.2004.01.005

[B7] Champion A. Jouannic S. Guillon S. Mockaitis K. Krapp A. Picaud A. (2004b). AtSGP1, AtSGP2 and MAP4K alpha are nucleolar plant proteins that can complement fission yeast mutants lacking a functional SIN pathway. *J. Cell Sci.* 15 4265–4275 10.1242/jcs.01200 15292395

[B8] Clough S. J. Bent A. F. (1998). Floral dip: a simplified method for *Agrobacterium*-media- ted transformation of *Arabidopsis* thaliana. *Plant J.* 16 735–743 10069079 10.1046/j.1365-313x.1998.00343.x

[B9] Cruz-Ramirez A. Lopez-Bucio J. Ramirez-Pimentel G. Zurita-Silva A. Sanchez-Calderon L. Ramirez-Chavez E. (2004). The xipotl mutant of *Arabidopsis* reveals a critical role for phospholipid metabolism in root system development and epidermal cell integrity. *Plant Cell* 16 2020–2034 15295103 10.1105/tpc.103.018648 PMC519195

[B10] Deveaux Y. Peaucelle A. Roberts G. R. Coen E. Simon R. Mizukami Y. (2003). The ethanol switch: a tool for tissue-specific gene induction during plant development. *Plant J.* 36 918–930 14675455 10.1046/j.1365-313x.2003.01922.x

[B11] Jouannic S. Champion A. Segiu-Simarro J. M. Salimova E. Picuad A. Tregear J. (2001). The protein kinases AtMAP3Kepsilon1 and BnMAP3Kepsilon1 are functional homologues of *S.pombe* cdc7p and may be involved in cell division. *Plant J.* 26 637–649 11489177 10.1046/j.1365-313x.2001.01065.x

[B12] Kulmburg P. Judewicz N. Mathieu M. Lenouvel F. Sequeval D. Felenbok B. (1992). Specific binding sites for the activator protein, ALCR, in the alcA promoter of the ethanol regulon of *Aspergillus nidulans*. *J. Biol. Chem.* 267 21146–21153 1400424

[B13] Laufs P. Coen E. Konenberger J. Traas J. Doonan J. (2003). Separable roles of UFO during floral development revealed by conditional restoration of gene function. *Development* 130 785–796 12506008 10.1242/dev.00295

[B14] Liljegren S. J. Ditta G. S. Eshed Y. Savidge B. Bowman J. L. Yanofsky M. F. (2000). SHATTERPROOF MADS-box genes control seed dispersal in *Arabidopsis*. *Nature* 404 766–770 10783890 10.1038/35008089

[B15] MAPK Group. (2002). Mitogen-activated protein kinase cascades in plants: a new nomenclature. *Trends Plant Sci.* 7 301–308 12119167 10.1016/s1360-1385(02)02302-6

[B16] Mellema S. Eichenberger W. Rawyler A. Suter M. Tadege M. Kuhlemeier C. (2002). The ethanolic fermentation pathway supports respiration and lipid biosynthesis in tobacco pollen. *Plant J.* 30 329–336 12000680 10.1046/j.1365-313x.2002.01293.x

[B17] Pickett M. Gwynne D. I. Buxton F. P. Elliot R. Davies R. W. Lockinton R. A. (1987). Cloning and characterization of the alcA gene of *Aspergillus nidulans*. *Gene* 51 217–216 3036652 10.1016/0378-1119(87)90310-6

[B18] Roberts G. R. Garoosi G. A. Koroleva O. Ito M. Laufs P. Leader D. J. (2005). The alc-GR system: a modified alc gene switch designed for use in plant tissue culture. *Plant Physiol.* 138 1259–1267 16010000 10.1104/pp.105.059659 PMC1176399

[B19] Roslan H. A. Salter M. G. Wood C. D. White M. R. Croft K. P. Robson F. (2001). Characterization of the ethanol-inducible *alc* gene-expression system in *Arabidopsis thaliana*. *Plant J.* 26 225–235 11722766 10.1046/j.1365-313x.2001.01146.x

[B20] Simanis V. (2003). Events at the end of mitosis in the budding and fission yeasts. *J. Cell Sci.* 116 4263–4275 14514882 10.1242/jcs.00807

[B21] Sparkes I. A. Brandizzi F. Slocombe S. P. El-Shami M. Hawes C. Baker A. (2003). An *Arabidopsis* pex10 null mutant is embryo lethal, implicating peroxisomes in an essential role during plant embryogenesis. *Plant Physiol.* 133 1809–1819 14576288 10.1104/pp.103.031252 PMC300734

[B22] Tadege M. Kuhlemeier C. (1997). Aerobic fermentation during tobacco pollen development. *Plant Mol. Biol.* 35 343–345 9349258 10.1023/a:1005837112653

[B23] Twell D. Yamaguchi J. McCormick S. (1990) Pollen-specific gene expression in transgenic plants: coordinate regulation of two different tomato gene promoters during microsporogenesis. *Development* 109 705–713 2401221 10.1242/dev.109.3.705

